# Complementary Regulation of Inflammatory, Catabolic, and PPARγ Signaling by Dexamethasone and Decanoic Acid in Donor-Specific Human Chondrocyte Models

**DOI:** 10.3390/biomedicines14092028

**Published:** 2026-09-09

**Authors:** Gregory W. Thomas, Jason Williams, Raphael Bar-Or, Melissa A. Hausburg, Kaysie Banton, David Bar-Or

**Affiliations:** 1 Trauma Research, Swedish Medical Center, 601 E. Hampden Ave. Suite 100, Englewood, CO 80113, USA; greg.thomas@trauma-research.com (G.W.T.); jason.williams@trauma-research.com (J.W.); rbaror@trauma-research.com (R.B.-O.); melissa.hausburg@trauma-research.com (M.A.H.); 2 Trauma Services, Swedish Medical Center, 501 E. Hampden Ave., Englewood, CO 80113, USA; banto001@gmail.com; 3 Trauma Research, Wesley Medical Center, 550 N. Hillside St, Wichita, KS 67214, USA; 4 Trauma Research, South Texas Health System, 301 W. Expy 83, McAllen, TX 78503, USA; 5 Trauma Research, Intermountain Health Lutheran Medical Center, 12911 W. 40th Ave., Wheat Ridge, CO 80401, USA

**Keywords:** osteoarthritis, dexamethasone, decanoic acid, peroxisome proliferator-activated receptor gamma, matrix metalloproteinase-13, prostaglandin E_2_

## Abstract

**Background/Objectives:** This study examined whether dexamethasone (Dex) and decanoic acid (DA) exert complementary effects on inflammatory signaling, catabolic gene expression, and peroxisome proliferator-activated receptor gamma (PPARγ) activation in human chondrocytes. **Methods:** Two donor-specific primary human chondrocyte models, each derived from a single knee donor, were used, in which cells were treated with Dex (≤0.1 µM), DA (≤500 µM), or combinations thereof. Acute Interleukin-1β(IL-1β)-induced PGE_2_ release was evaluated in normal donor cells, whereas prolonged transcriptional responses and PPARγ DNA-binding activity were evaluated in OA donor cells. IL-1β-induced prostaglandin E_2_ (PGE_2_) release was measured by competitive ELISA after 24 h. *Glyceraldehyde-3-phosphate dehydrogenase*-normalized transcription of *Collagen type II alpha 1*, *Collagen type I alpha 1*, *Aggrecan*, *SRY-Box Transcription Factor 9*, *Runt-Related Transcription Factor 2*, and *Matrix Metalloproteinase 13 (MMP13)* was assessed by qRT-PCR at 7, 10–14, and 20–28 days. In addition, PPARγ DNA-binding activity was measured after 1 week. Interactions were evaluated by Loewe additivity and highest single agent (HSA) analyses. **Results:** Dex and DA each reduced PGE_2_ release, whereas co-treatment increased potency and maximal inhibition, with a Loewe combination index of 0.4 at 1 nM Dex plus 19 µM DA. In the temporal analysis, Dex plus DA showed a positive *MMP13* ΔCt difference versus Dex at 10–14 days (2.42 ± 2.14; *p* = 0.065) and a more consistent difference at 20–28 days (1.11 ± 0.25; *p* = 0.016). This agreed with fixed-dose HSA findings at 14 and 28 days (ΔHSA ≈ −75 for 0.1 µM Dex plus 100 µM DA versus Dex). Co-treatment was also associated with increased PPARγ activation beyond the HSA reference (ΔHSA ≈ 0.09 for 0.1 µM Dex plus 100–250 µM DA versus Dex). **Conclusions:** Dex plus DA co-treatment produced complementary effects on acute PGE_2_ inhibition, as well as late-onset *MMP13* suppression and PPARγ activity in the donor-specific chondrocyte models studied. These hypothesis-generating findings provide an in vitro rationale for future evaluation of this combination as a corticosteroid-sparing strategy.

## 1. Introduction

Osteoarthritis (OA) is the most common chronic joint disease and a leading cause of disability worldwide, affecting up to 40% of adults over the age of 60 [1,2]. Rather than a purely degenerative disease, OA is increasingly recognized as a chronic inflammatory disorder characterized by synovial activation, prostaglandin production, and the induction of cartilage-degrading proteases [3,4]. Thus, an ideal therapeutic for OA would restore joint homeostasis and improve function by targeting the underlying inflammation while preserving the immune system’s natural healing properties.

Intra-articular corticosteroids are widely used in the treatment of OA because they provide rapid and often substantial symptomatic relief; however, their benefits are transient, and concerns have emerged that repeated or high-dose exposure may accelerate structural deterioration [5]. Furthermore, experimental models demonstrate dexamethasone (Dex)–associated proteoglycan loss, altered subchondral bone quality, and increased chondrocyte apoptosis [6,7]. Moreover, persistent steroid use may compromise healing and increase the risk of wound complications [7]. Clinical guidance, therefore, emphasizes minimizing cumulative steroid exposure.

One approach to balancing efficacy with structural safety is to engage complementary pathways that modulate inflammation and promote resolution. Peroxisome proliferator-activated receptor gamma (PPARγ) is a transcription factor that integrates lipid signaling and inflammatory transcriptional control [8] and is implicated in cartilage homeostasis and OA progression [9,10,11]. Activation of PPARγ represses NF-κB and AP-1 signaling, resulting in the decreased production of inflammatory cytokines such as TNFα and IL-6 [12,13], and contributes to tissue repair [14]; potentially through the anti-inflammatory and anti-fibrotic activities of endogenous ligands such as 15-Deoxy-Δ-12,14-Prostaglandin J_2_ [15]. Importantly, loss of PPARγ activity in cartilage promotes OA-like pathology [9]. Evidence also suggests that glucocorticoid and PPARγ signaling intersect mechanistically. Dex can upregulate PPARγ expression [16], while PPARγ activation attenuates glucocorticoid-associated metabolic stress [17]. Medium-chain fatty acids, particularly decanoic acid (DA), function as selective weak PPARγ ligands [18], stabilizing receptor conformation while avoiding full adipogenic activation [18,19]. Based on these findings, Dex and PPARγ agonism may complement each other in the treatment of OA.

It is now well accepted that OA pathogenesis involves persistent inflammatory signaling, dysregulated tissue remodeling, and the failure of endogenous regulatory pathways that normally restore joint homeostasis. Therapeutic approaches capable of simultaneously suppressing inflammatory mediators while reinforcing protective regulatory programs may therefore provide advantages over single-target interventions. Because Dex and DA influence distinct but potentially complementary signaling pathways, we hypothesized that combined treatment would produce broader biological effects than either compound alone. To test this hypothesis, we evaluated acute inflammatory responses through IInterleukin-1β(IL-1β)-induced prostaglandin E_2_ (PGE_2_) synthesis, chronic catabolic regulation through *matrix metalloproteinase-13* (*MMP13*) transcription, and regulatory signaling via PPARγ activation in primary human chondrocytes.

## 2. Materials and Methods

### 2.1. Cell Culture and Passaging

Primary human synovial-joint chondrocytes from a single normal knee joint donor (HSC-N; catalog# CDD-H-2610-N, donor ID# H-1494, male, 52 years of age) and a single donor with knee OA (HSC-OA; catalog# CDD-H-2610-OA, donor ID# H-1512, female, 79 years of age) were obtained commercially from Articular Engineering (Northbrook, IL, USA). These preparations constituted the donor-specific HSC-N and HSC-OA experimental models used throughout the study. All other culturing reagents were purchased from ThermoFisher Scientific (Waltham, MA, USA) unless otherwise stated. For experiments, cells were seeded at 3000 cells/cm^2^ in the tissue culture plates described below and then expanded in DMEM/F12 containing 20% FBS at 37 °C and 5% CO_2_. When 100% confluent, the cultures were equilibrated for 24 h in DMEM/F12 containing 5% FBS before treatment, with treatments performed in the 5% FBS medium. HSC-N were utilized at passages 2–3 and HSC-OA at passages 5–7. For these studies, DA refers to decanoate administered as commercially sourced sodium decanoate (Sigma-Aldrich, St. Louis, MO, USA), which was prepared directly in buffered culture medium with BSA serving as a fatty-acid carrier.

### 2.2. Inflammatory Stimulation and PGE_2_ Measurements in HSC-N

Acute-phase inflammatory stimulation and PGE_2_ release were measured in HSC-N cultures. In brief, cells were seeded into 96-well tissue culture plates as described above and then exposed to dexamethasone sodium phosphate (Dex; 0.01–100 nM in a 10-fold serial dilution), decanoic acid (DA; 6–500 µM in a 3-fold serial dilution), or the Dex dilution series with DA incorporated at set amounts across the range (1, 2, 10, 12.5, 19, or 50 µM DA), and then activated with IL-1β at a final concentration of 0.1 ng/mL. These concentrations were selected from preliminary concentration-response studies to span weak to near-saturating inhibition responses. Supernatants were collected 24 h after stimulation, and PGE_2_ was measured via competitive ELISA (Cayman Chemical, Ann Arbor, MI, USA).

### 2.3. qRT-PCR Analysis of Chondrocyte Markers in HSC-OA

Long-term transcriptional regulation was measured in resting HSC-OA cultures using quantitative real-time PCR. In brief, cells were seeded into 24-well tissue culture plates and maintained under treatment for up to 28 days (0.1 µM Dex, 50 or 100 µM DA, or in the corresponding combination), with the medium refreshed every 2–3 days. Total RNA was isolated using Qiagen RNeasy plus columns (Germantown, MD, USA) at 7, 10–14, and 20–28 days, and amplification from complementary DNA was performed for *Glyceraldehyde-3-phosphate dehydrogenase* (*GAPDH*; GeneGlobe ID# PPH00150F), *Collagen type II alpha 1* (*Col2a1*; GeneGlobe ID# PPH02134F), *Collagen type I alpha 1* (*Col1a1*; GeneGlobe ID# PPH01299F), *Aggrecan* (*ACAN*; GeneGlobe ID# PPH06097E), *SRY-Box Transcription Factor 9* (*SOX9*; GeneGlobe ID# PPH02125A), *Runt-Related Transcription Factor 2* (*RunX2*; GeneGlobe ID# QT00020517), and *MMP13 *(GeneGlobe ID# PPH00121B) using validated RT^2^ qPCR Primer Assays (catalog# 330001) and reagents from Qiagen. *GAPDH*-normalized ΔCt and corresponding ΔΔCt values were then calculated, with temporal treatment responses presented as mean ± SD ΔΔCt. qRT-PCR amplification and fluorescence detection were performed using a LightCycler^®^ 480 Instrument II (Roche Diagnostics GmbH, Mannheim, Germany). Amplifications were performed by heating 20 µL reaction volumes to 95 °C for 10 min, then conducting 45 cycles of 95 °C for 15 s followed by 60 °C for 1 min. Temporal qRT-PCR results were grouped into Day 7, Day 10–14, and Day 20–28 culture-day windows to capture early, intermediate, and later transcriptional responses across the prolonged culture period. Closely spaced harvest days were combined within each window to preserve the intended temporal progression of the experiments while allowing consistent comparison across independently performed cultures.

### 2.4. PPARγ Activity in HSC-OA

Regulatory PPARγ activation was then assessed in resting HSC-OA cultures. In brief, cells were seeded into 10 cm^2^ dishes as described above, treated for the times indicated with 0.1 µM Dex, 100 µM DA, 250 µM, or combinations thereof, and nuclear protein extracts were isolated. Rosiglitazone (Rosig; 10 µM) obtained from Sigma-Aldrich (St. Louis, MO, USA) served as a positive control for these experiments. Nuclear DNA-binding activity was then measured using a capture assay from Active Motifs (Carlsbad, CA, USA).

### 2.5. Statistical Analysis

Statistical analyses were performed in Python 3.12.3 using scientific computing libraries (NumPy 2.1.3 [20], SciPy 1.14.1 [21], and pandas 2.2.3 [22]).

For these investigations, we employed a replication strategy in which each reported experimental sample represented an independently established culture preparation that was separately treated and processed through the corresponding assay workflow. For qRT-PCR, RNA isolation, cDNA synthesis, and PCR amplification were likewise performed independently for each preparation.

For PGE_2_ release experiments, percent inhibition was calculated relative to stimulated controls. Loewe combination indices were then derived from fitted dose–response curves, with uncertainty estimated using 350 nonparametric bootstrap resamples of independently established experimental samples to calculate 95% confidence intervals. Relative potency of Dex in the presence of fixed DA concentrations was estimated by parallel-line analysis using PLA 3.0 (Stegmann Systems GmbH, Rodgau, Germany), with an on-plate Dex reference included in each independent experiment. Associations between DA concentration and relative potency were evaluated by log-log regression, Pearson’s *r*, Spearman’s ρ, and R^2^. Detailed equations and calculation procedures are provided with the deposited source data.

For qRT-PCR experiments, target-gene expression was normalized to GAPDH as ΔCt = Ct_target − Ct_GAPDH. For within-experiment relative-expression analysis, ΔΔCt was calculated as ΔCt_treatment − ΔCt_Medium and relative expression as 2^−^^ΔΔCt^. For *MMP13* fold-regulation presentation, values ≥1 were reported directly, whereas values <1 were expressed as the negative reciprocal [−1/(2^−ΔΔCt^)]; thus, negative values indicate the magnitude of downregulation rather than negative expression. Temporal qRT-PCR analyses were performed at the independent experiment/time-point level using within-experiment treatment contrasts. For N ≥ 3, contrasts were evaluated against zero using two-sided one-sample *t*-tests with 95% confidence intervals; N < 3 comparisons were considered descriptive. Holm-adjusted *p* values were calculated across testable targets within each temporal interval, separately for each treatment-contrast family. Detailed analysis procedures and scripts are provided with the deposited source data.

*MMP13* relative expression and PPARγ activity data were tested using one-sample, two-tailed *t*-tests against a hypothetical value of 1.0 or by Welch’s two-sample *t*-tests. Interaction effects in single-dose *MMP13* relative expression and PPARγ activity assays were evaluated using a variance-aware Highest Single Agent (HSA) analysis, with uncertainty estimated via nonparametric bootstrap resampling (1000 iterations) to derive 95% confidence intervals. HSA was interpreted as determining whether the combination exceeded the best-performing single agent. Figures were generated using Matplotlib 3.9.2 [23].

## 3. Results

### 3.1. DA Enhances Dex-Mediated Inhibition of IL-1β-Induced PGE_2_ in HSC-N

**Figure 1:**
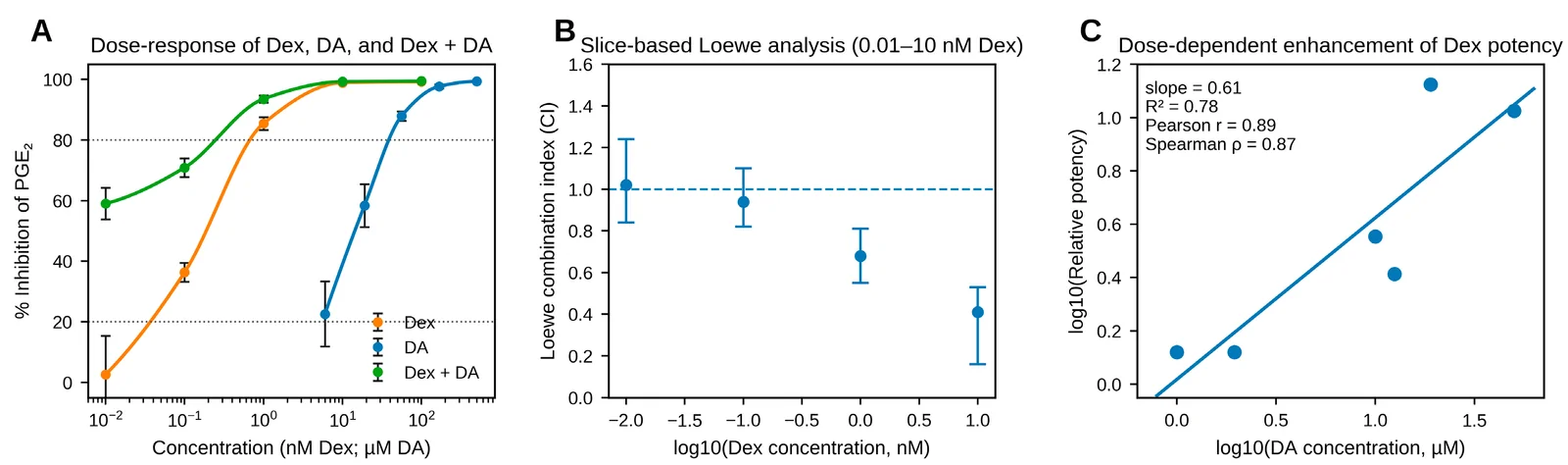
DA enhances Dex-mediated inhibition of IL-1β-induced PGE_2_ release from HSC-N. These experiments were performed using primary HSC-N derived from a single normal knee donor, with experimental observations obtained from independently cultured and treated preparations. Panels (**A**,**B**) each contained n = 3 independently cultured and treated preparations per concentration in the representative experiment. Panel (**C**) summarizes N = 6 independent experiment-days, with one fixed DA concentration evaluated per experiment and n = 3 independently established preparations per concentration point. (**A**) PGE_2_ inhibition dose–response curves for DA (6–500 µM), Dex (0.01–100 nM), and Dex + 19 µM DA. After 24 h, the inhibition of PGE_2_ release from IL-1β-stimulated HSC-N was calculated compared to controls. Data are presented as mean ± SD fractional inhibition from a representative experiment performed in triplicate across independent culture preparations or experimental replicates from the same donor. (**B**) Slice-based Loewe analysis of the representative dataset. Compound interaction was evaluated using a restricted, slice-based Loewe additivity analysis applied to the representative dataset in (**A**). Single-agent dose–response curves were used to estimate equivalent doses at matched effect levels, and combination index (CI) values were calculated across Dex concentrations from 0.01 to 10 nM in the presence of 19 µM DA. Bootstrap confidence intervals (350 resamples) are shown. CI values were interpreted primarily within the EC20–EC80 response range to protect against saturation effects. (**C**) Relative potency (REP) was determined by parallel line analysis from six independent Dex dose–response experiments performed on separate days, using paired on-plate Dex-alone and Dex + DA curves. Data are presented as a regression analysis of log10 REP versus log10 dose of DA incorporated into the Dex dose–response curves. REP increased with DA concentration, indicating a dose-dependent enhancement of Dex potency across independent culture preparations or experimental replicates from the same donor.

To determine whether DA enhances the ability of Dex to regulate acute inflammatory signaling, we used IL-1β-stimulated HSC-N as an inducible inflammatory model and evaluated the effects of each compound alone and in combination on PGE_2_ production (Figure 1A). Cells were treated with DA (6–500 µM), Dex (0.01–100 nM), and a Dex series containing a consistent concentration of DA (19 µM) across the range. The cultures were then stimulated with 0.1 ng/mL IL-1β for 24 h; PGE_2_ release was quantified, and dose–response curves were generated as percent PGE_2_ inhibition calculated versus controls. We found that DA inhibited PGE_2_ release in a dose-dependent manner, with 58% suppression observed at 19 µM and 95% at concentrations ≥167 µM. Furthermore, Dex dose-dependently inhibited PGE_2_, ranging from a 10% reduction at 0.01 nM to 99% inhibition at ≥10 nM. In the presence of 19 µM DA, enhanced Dex inhibition was observed from 36% to 71% at 0.1 nM and from 85% to 94% at 1 nM, indicating an apparent increase in potency.

Based on the observed leftward shift in the combined dose–response curve, interaction between Dex and DA was explored using a slice-based Loewe analysis of the representative 19 µM DA dataset (Figure 1B). Loewe analysis assesses dose additivity by comparing observed combination responses to expected additive effects, with the combination index (CI) quantifying deviations from additivity (CI = 1 indicates additivity; CI < 1 indicates synergy; CI > 1 indicates antagonism). Single-agent dose–response fits from the representative dataset were used to define the expected response assuming additivity (CI = 1) across the response range. Observed Dex + DA responses were then compared with these expected additive inhibitions using the DA dose (“slice”) employed in the experiment. At 0.01 nM Dex, CI values were centered near additivity (median CI ≈ 1.0). CI values decreased at intermediate Dex concentrations (0.1–1 nM), reaching approximately 0.4 at 1 nM Dex, with bootstrap confidence intervals that did not include CI = 1, consistent with potential synergy. Importantly, our interpretation focused primarily on the EC20–EC80 response range. At higher Dex concentrations, near-maximal inhibition impedes analysis, rendering CI estimates unreliable and therefore not presented.

As a complementary assessment of potency enhancement, relative potency was then determined from six independent Dex dose–response experiments performed on separate days, each containing a paired on-plate Dex reference and Dex + DA curve (Figure 1C). REP was positively associated with DA concentration, with a log-log regression slope of 0.61 (95% CI, 0.16–1.05; R^2^ = 0.78), Pearson’s *r* = 0.89, and Spearman’s ρ = 0.87. This pattern was concordant with the Loewe analysis; however, the Loewe and REP analyses should be considered exploratory because they represent a single fixed DA slice, and DA activity was not validated across days of the experiment.

### 3.2. DA and Dex-Induced Transcriptional Regulation as Well as Enhanced Long-Term Suppression of MMP13 in HSC-OA by Combinatorial Treatment

While the previous assays focused on acute, inducible inflammatory responses, HSC-OA cultures were then used to examine longer-term transcriptional responses in an OA-derived cellular background and determine whether the investigational compounds could preserve or restore cartilage-related gene-expression patterns. Chondrogenic regulators (*SOX9*), structural matrix components (*COL2A1* and *ACAN*), and fibrogenic, hypertrophic, or catabolic markers (*COL1A1*, *RUNX2*, and *MMP13*) were analyzed using *GAPDH*-normalized ΔCt values to assess normalized transcript abundance and the ΔΔCt method to determine relative expression changes compared with the corresponding medium control. It is important to note that no overt cytotoxicity was observed during the prolonged culture period as assessed by LDH release or MTS conversion (Representative MTS data provided in Table S5). This extended kinetic window enabled assessment of sustained transcriptional regulation beyond immediate, short-term inflammatory responses.

**Figure 2:**
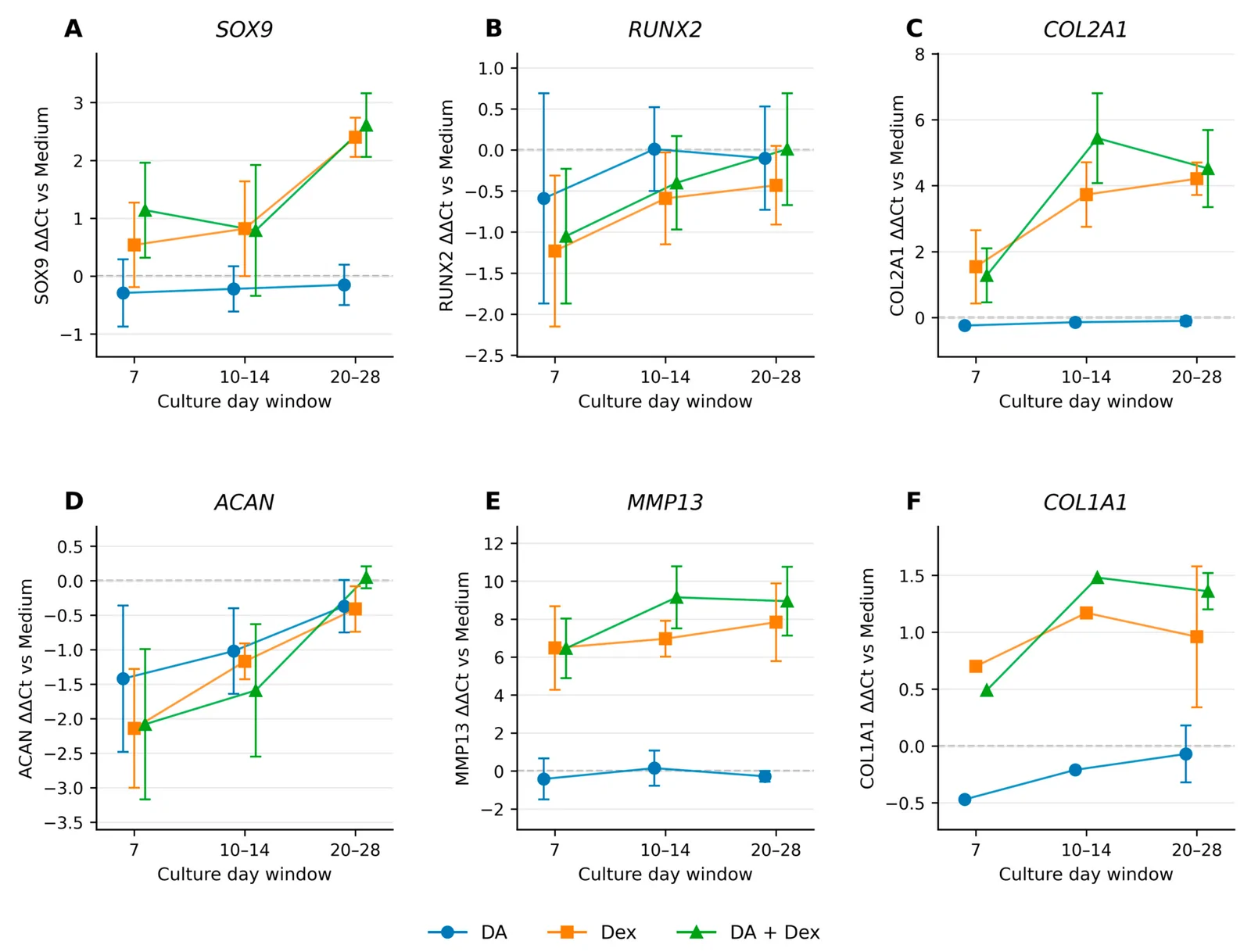
Temporal transcriptional responses of chondrocyte markers following DA, Dex, or DA + Dex treatment in HSC-OA. Treatment-versus-Medium ΔΔCt responses are shown for (**A**) *SOX9*, (**B**) *RUNX2*, (**C**) *COL2A1*, (**D**) *ACAN*, (**E**) *MMP13*, and (**F**) *COL1A1* across the Day 7, Day 10–14, and Day 20–28 culture-day windows. Positive ΔΔCt values indicate lower GAPDH-normalized transcript abundance relative to Medium, whereas negative values indicate higher relative abundance. Data are shown as mean ± SD ΔΔCt where calculable.

A temporal ΔCt analysis demonstrated selective, time-dependent regulation across the six chondrocyte markers (Figure 2; Supplementary Tables S1 and S2). Figure 2 presents treatment-versus-medium ΔΔCt responses across Day 7, Day 10–14, and Day 20–28 culture-day windows. DA alone produced comparatively limited responses, whereas Dex and DA + Dex produced the largest later changes in *MMP13* and *COL2A1*. At Day 10–14, *MMP13* ΔΔCt was 6.97 ± 0.94 with Dex (N = 5; *p* < 0.001; Holm-adjusted *p* < 0.001) and 9.15 ± 1.64 with DA + Dex (N = 6; *p* < 0.001; Holm-adjusted *p* < 0.001), indicating lower normalized *MMP13* transcript abundance relative to medium. Dex also altered *SOX9* at Day 20–28 (ΔΔCt, 2.40 ± 0.34; *p* = 0.007; Holm-adjusted *p* = 0.026) and *ACAN* at Day 10–14 (ΔΔCt, −1.17 ± 0.26; *p* = 0.016; Holm-adjusted *p* = 0.048). Complete numerical treatment-versus-medium results are provided in Supplementary Table S1, with the underlying experiment-level ΔCt values provided in Supplementary Table S2.

The treatment-versus-medium ΔΔCt profiles were further evaluated by direct DA + Dex-versus-Dex comparisons (Supplementary Table S1). For *MMP13*, the ΔCt difference was 2.42 ± 2.14 at Day 10–14 (95% CI, −0.24 to 5.07; N = 5; *p* = 0.065; Holm-adjusted *p* = 0.259) and 1.11 ± 0.25 at Day 20–28 (95% CI, 0.50 to 1.72; N = 3; *p* = 0.016; Holm-adjusted *p* = 0.080). The confidence interval excluded zero at Day 20–28 but included zero at Day 10–14. A combination-associated difference was also observed for *COL2A1* at Day 10–14 (1.71 ± 0.46 ΔCt; 95% CI, 0.98 to 2.44; N = 4; *p* = 0.005; Holm-adjusted *p* = 0.025). No corresponding DA + Dex-versus-Dex effect was detected for *SOX9*, *RUNX2*, or *ACAN*, while *COL1A1* remained descriptive because of limited experiment-level coverage. Day 7 comparisons were also considered descriptive because only two independent experiment/time-point contrasts were available. Overall, these results support selective combination-associated transcriptional effects, with *MMP13* showing the clearest later response for focused evaluation.

Because GAPDH served as the reference gene, raw Ct behavior was also evaluated (Supplementary Table S3). GAPDH showed modest treatment-associated variation; however, DA + Dex did not differ from Dex overall (mean difference, −0.04 Ct; 95% CI, −0.42 to 0.34; *p* = 0.824; Holm-adjusted *p* = 0.888), and no consistent temporal combination-associated difference was observed. Thus, DA + Dex-versus-Dex comparisons were not associated with a corresponding overall change in GAPDH Ct.

Taken together with the acute PGE_2_ findings, these data support a temporally layered response in which DA + Dex enhances early control of extracellular PGE_2_ and produces delayed, sustained regulation of selected catabolic and matrix-remodeling genes. *MMP13* demonstrated the clearest later combination-associated advantage and therefore remained the most appropriate target for focused mechanistic analysis.

**Figure 3:**
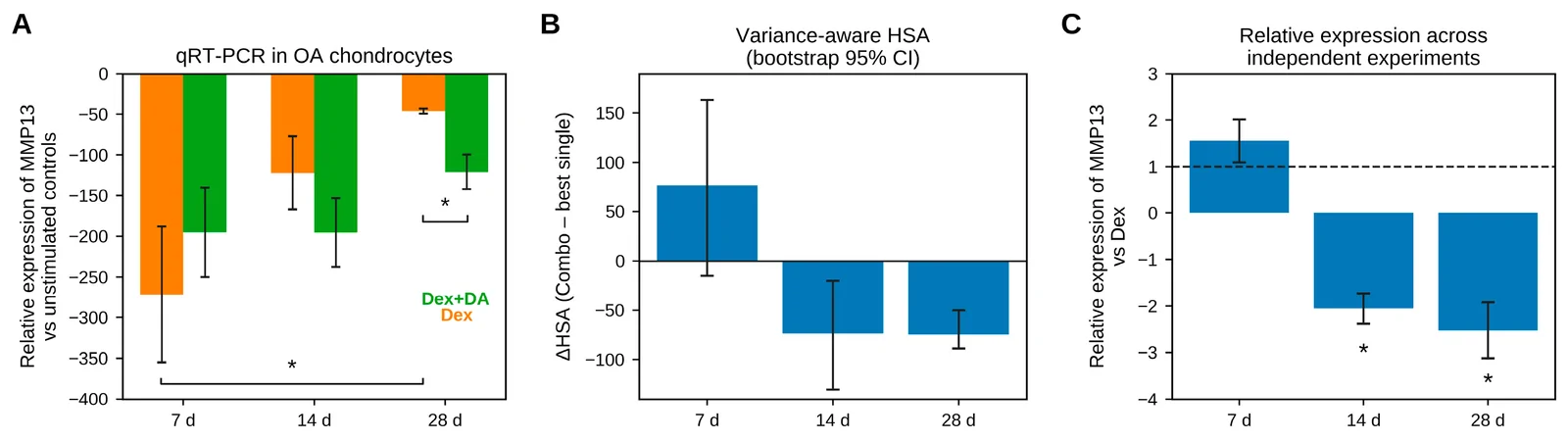
DA enhances Dex-mediated suppression of *MMP13* expression in long-term cultures of HSC-OA. These experiments were performed using primary HSC-OA derived from a single knee OA donor, with experimental observations obtained from independently cultured, treated, and processed preparations. Panels (**A**,**B**) included n = 3 independently cultured, treated, and processed preparations per treatment at each time point. Panel (**C**) summarizes N = 4, 5, and 4 independent experiment-level values at Days 7, 14, and 28, respectively. In addition, all RNA isolation, first-strand cDNA synthesis, and qRT-PCR were performed separately for each preparation. Fold regulation was derived from 2^−ΔΔCt^ relative expression. Values below 1 were expressed as the negative reciprocal; thus, negative values indicate the magnitude of downregulation relative to the corresponding within-experiment Medium control and do not represent negative expression. (**A**) Relative expression of *MMP13* determined by quantitative RT-PCR (ΔΔCt), normalized to GAPDH and unstimulated controls, following treatment with 0.1 µM Dex, 100 µM DA, or Dex + DA for 7, 14, or 28 days. Data presented as mean ± SD of relative expression vs. unstimulated controls from a representative experiment performed in triplicate (n = 3). * = *p*-value ≤ 0.05 by Welch’s *t*-test. (**B**) Variance-aware Highest Single Agent (HSA) analysis of *MMP13* expression applied to the representative data set in (**A**). ΔHSA values represent the difference between the combination and the most suppressive single agent at each time point; negative values indicate greater suppression by the combination than by the most suppressive single agent. Error bars denote 95% bootstrap confidence intervals (1000 resamples). (**C**) Relative expression of *MMP13* normalized to Dex within independent experiments (REP-based normalization), illustrating reproducibility across independent culture preparations or experimental replicates from the same donor performed on different days. Data presented as mean ± SD of relative expression vs. Dex-treated controls (n = 4 for Day 7 and Day 28, n = 5 for Day 14). * = *p*-value ≤ 0.05 by one-sample *t*-tests (hypothetical value of 1.0).

To examine the identified *MMP13* pattern under more standardized experimental conditions, focused Day 7, Day 14, and Day 28 experiments were evaluated separately from the broader temporal analysis. Figure 3A shows *MMP13* relative expression in a representative fixed-dose experiment, calculated by the ΔΔCt method following normalization to *GAPDH* and the corresponding unstimulated control, after treatment with 0.1 µM Dex, 100 µM DA, or Dex + DA for 7, 14, and 28 days. DA alone had minimal effect throughout the time course, whereas Dex initially produced robust inhibition of *MMP13* expression that diminished over time. In contrast, Dex + DA maintained greater MMP13 downregulation than Dex alone at the later Day 14 and Day 28 time points, corresponding to approximately 195 ± 55-fold lower expression compared with 121 ± 42-fold lower expression with Dex alone. These representative relative-expression findings were consistent with the matched temporal DA + Dex-versus-Dex analysis described above, which showed positive combination-associated ΔCt differences at both later intervals, with the Day 20–28 95% CI excluding zero. Together, these findings suggest that DA reinforces the long-term transcriptional regulation of *MMP13* during glucocorticoid treatment and may help maintain biological responses that diminish with Dex alone.

A variance-aware Highest Single Agent (HSA) analysis was then used to determine whether *MMP13* transcript inhibition by the Dex + DA combination exceeded Dex treatment alone (Figure 3B). HSA measures whether the combined treatment outperforms the best-performing single agent at the same dose, in this case, Dex monotherapy. After 7 days, the ΔHSA score fails to indicate a difference between Dex and combination treatment; however, after 14 and 28 days of DA + Dex treatment, negative ΔHSA scores indicate that the combination more effectively suppresses *MMP13* transcript compared to Dex alone.

To test robustness and control for inter-assay variability, we calculated the relative expression of *MMP13*, normalized to Dex, across independent experiments. Figure 3C shows the average relative expression of independent culture preparations or experimental replicates from the same donor performed on different days (n = 4 or 5). At 14 and 28 days, Dex + DA treatment reduced expression when compared to Dex alone. As a result, despite the expected heterogeneity of long-term OA chondrocyte cultures, the combination of DA + Dex consistently suppresses *MMP13* transcript.

### 3.3. Dex Amplifies DA-Driven PPARγ Activation in HSC-OA

Loss of PPARγ activity may play a role in the etiology of OA, and upregulation is beneficial in experimental models [9,10,11]. Furthermore, DA is a known ligand for PPARγ with a unique activation profile [18]. Moreover, Dex has been shown to increase PPARγ expression in fibroblast-like cells [16]. Thus, we hypothesized that the combination treatment of Dex + DA would promote PPARγ activation in human chondrocytes.

**Figure 4:**
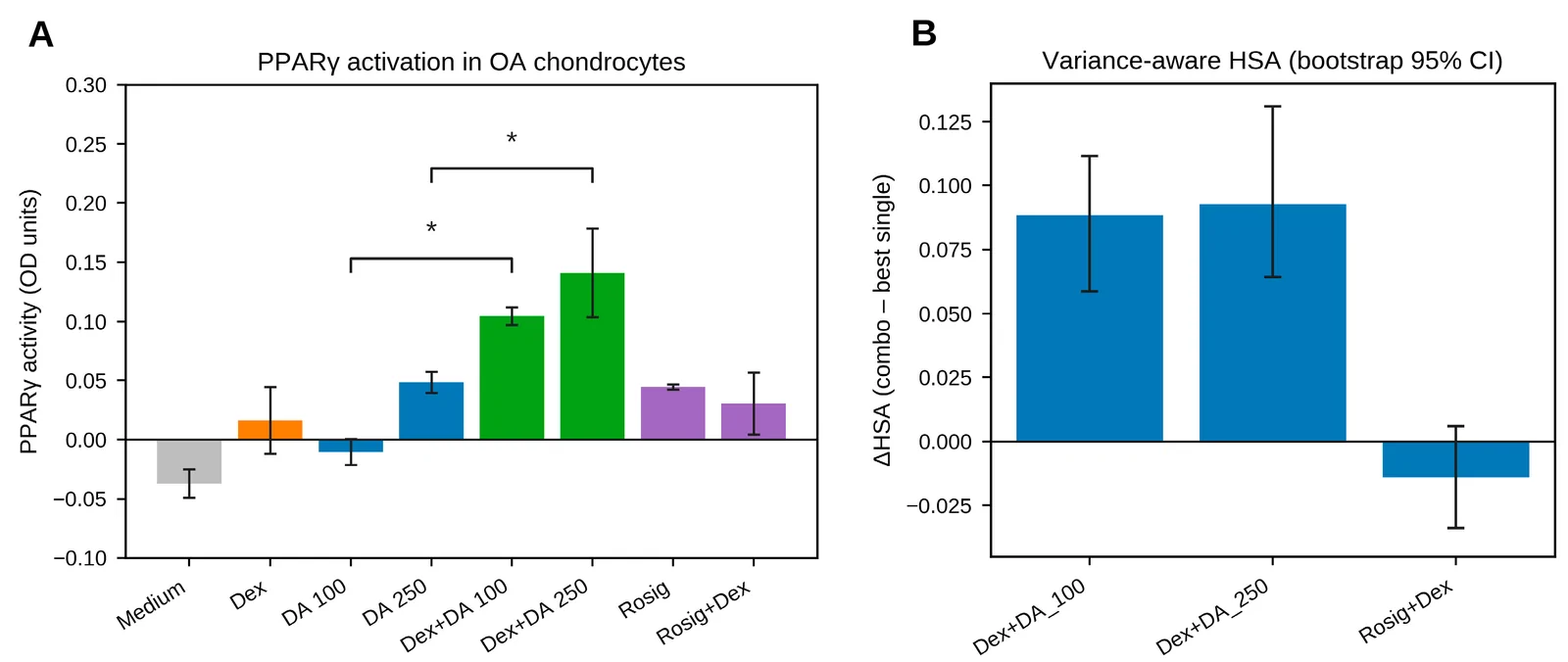
Dex enhances DA-mediated activation of PPARγ in HSC-OA. These experiments were performed using primary HSC-OA derived from a single knee OA donor. Each experimental preparation was independently cultured, treated, harvested, processed for nuclear extraction, and assayed for PPARγ DNA-binding activity. In panel (**A**), n = 3 independently cultured and processed preparations were analyzed for the Medium, Dex, DA, and DA + Dex conditions, whereas n = 2 preparations were analyzed for rosiglitazone and rosiglitazone + Dex. Panel (**B**) was calculated from the same preparation-level observations. (**A**) PPARγ transcription factor activity measured in nuclear extracts from human OA chondrocytes after 7 days of treatment with 0.1 µM Dex, 100 and 250 DA µM, Dex + DA combinations, 10 µM rosiglitazone (Rosig), or Rosig + Dex under unstimulated conditions. Data are presented as the mean OD ± SD (N = 3). * = *p*-value ≤ 0.05 by Welch’s *t*-test. (**B**) Variance-aware Highest Single Agent (HSA) analysis applied to the representative data set in (**A**). ΔHSA values represent the difference between combination and best single-agent effects; positive values indicate that activated PPARγ exceeds the best-performing single agent. Error bars denote 95% bootstrap confidence intervals (1000 resamples).

To test this hypothesis, PPARγ DNA-binding activity was measured using an assay that captures active PPARγ from nuclear extracts with oligonucleotides containing specific response-element sequences (Figure 4A). HSC-OA were treated with 0.1 µM Dex, 100 or 250 µM DA, or combinations thereof, and nuclear protein was extracted after one week. Rosiglitazone was used as a control (10 µM) for 2 h in resting cultures or in cells treated with Dex alone for a week. In the individual test compound groups, only 250 µM DA produced a detectable signal above the background. Interestingly, the Dex + DA samples showed a dose-dependent increase in activity, with 100 µM exhibiting an OD of 0.105 ± 0.008 and 250 µM at 0.141 ± 0.037. Rosiglitazone-treated samples similarly showed significant increases in OD (0.045 ± 0.009 and 0.031 ± 0.026, respectively, for alone or following 1 week Dex).

To determine whether the combination exceeded additivity, a variance-aware HSA analysis was again performed on these data (Figure 4B). For both Dex + 100 µM DA and Dex + 250 µM DA, positive ΔHSA values showed that the combination DA + Dex treatment exceeded the ability of DA alone to promote PPARγ DNA-binding activity. In contrast, the ΔHSA score of Rosig + Dex shows that this combination treatment was unable to activate PPARγ more than the effect of rosiglitazone alone. These findings demonstrate increased PPARγ DNA-binding activity following DA + Dex treatment under the conditions evaluated, which may be independent of a simple increase in expression caused by steroid therapy.

## 4. Discussion

The present study demonstrates that Dex combined with DA exerts complementary regulation across inflammatory, catabolic, and nuclear receptor pathways in human chondrocytes. Three consistent observations support this conclusion: enhanced suppression of IL-1β-induced PGE_2_, sustained repression of *MMP13* transcription during prolonged culture, and markedly increased PPARγ DNA-binding activity when cells were treated with the combination DA + Dex compared with either agent alone. Several mechanisms or modes of action associated with DA have recently been identified, which may help explain these results. Research by our lab shows that in vitro DA administration causes both the production of pro-resolving lipid mediators such as maresin and the inhibition of Multiple Drug Resistance Protein 4 (MRP4) (Unpublished data). We hypothesize that these newly discovered DA effects contribute to the complementary and combination-associated responses observed with glucocorticoids in both acute-phase and long-term chondrocyte OA culture models.

One of our key findings was that DA dose-dependently increased the relative potency of Dex as measured by inhibition of IL-1β-induced PGE_2_ release after 24 h. Slice-based Loewe analysis revealed a concentration-dependent deviation from additivity between Dex and DA, suggesting a modest trend toward synergy within the EC20–EC80 window, where equivalent-dose analysis is most stable. Despite the expected inter-assay variability that is inherent in biological assays, the consistency of the REP trend (i.e., R^2^ ≈ 0.78; Pearson r ≈ 0.88; Spearman ρ ≈ 0.87) supports the robustness of this interaction. Together, these findings indicate that DA augments Dex efficacy primarily within the linear portion of the dose–response curve. This pattern is consistent with a greater-than-expected interaction that becomes more pronounced as Dex efficacy increases, but before full saturation is reached.

Mechanistically, this may reflect convergent effects on COX-2 expression, prostaglandin synthesis, or efflux regulation. Dex targets COX-2 in a multifaceted fashion by downregulating transcriptional regulation, destabilizing mRNA, and inhibiting signaling pathways that induce expression [24,25]. In addition, MRP4 is a transmembrane transporter that helps regulate cellular function by pumping organic anions, drugs, cellular waste products, and signaling molecules such as cAMP and prostaglandins [26]. Thus, combined Dex and DA therapy could enhance PGE_2_ inhibition by both reducing inducible COX-2 and retaining its products intracellularly. From a translational standpoint, the observation raises the possibility that lower Dex concentrations could achieve comparable inflammatory control when administered with DA, providing an in vitro rationale for testing a potential steroid-sparing strategy.

Inhibition of PGE_2_ is clinically relevant because prostaglandins and eicosanoids play a significant role in the acute phase of inflammation by driving neutrophil recruitment, pain, and swelling [27]. These represent important protective immune responses that are usually self-limiting but can evolve into chronic inflammation if dysregulated [27]. As for OA, inflamed joint tissues overexpress COX-2, resulting in elevated PGE_2_ production, and NSAIDS are frequently prescribed to alleviate symptoms [28]. Alternatively, low doses of glucocorticoids, used for brief periods, can control inflammation without compromising the healing process. For example, Dex has been shown to beneficially modify the lipid mediator signature of proinflammatory innate immune cells associated with acute hyper-inflammation in favor of pro-resolving patterns [29]. Providing a means to reduce glucocorticoid dosing may help limit the overall immune response, while preserving the protective and immune-switching functions of lipid mediators, improving clinical outcomes.

We also found that extended Dex and DA treatment provided a complementary view on the suppression of MMP13, a collagenase associated with early-onset OA [30]. In the representative fixed-dose experiment, Dex initially induced substantial MMP13 suppression, consistent with its known inhibition of MMP13 expression in chondrocytes [31], but this response diminished over time. Co-administration with DA maintained greater downregulation at 14 and 28 days. In the temporal analysis, the DA + Dex-versus-Dex difference was 2.42 ± 2.14 ΔCt at Day 10–14 (95% CI, −0.24 to 5.07; N = 5; *p* = 0.065; Holm-adjusted *p* = 0.259) and 1.11 ± 0.25 ΔCt at Day 20–28 (95% CI, 0.50 to 1.72; N = 3; *p* = 0.016; Holm-adjusted *p* = 0.080). The combination-associated difference remained directionally positive at both later intervals, although neither comparison remained significant after correction across the broader marker screen. These temporal findings were directionally concordant with the focused fixed-dose *MMP13* experiments evaluated separately at Day 14 and Day 28.

Importantly, the broader transcriptional response was selective rather than uniformly anabolic or chondroprotective. DA alone produced limited changes across most markers. Dex and DA + Dex suppressed *SOX9* and *COL2A1* at later intervals, with an additional matched Day 10–14 *COL2A1* effect. Reduced *RUNX2* could be consistent with restraint of hypertrophic differentiation, but *RUNX2* did not show a consistent treatment-versus-medium response. *ACAN* showed treatment-associated induction without a matched combination advantage, whereas *COL1A1* showed time-dependent suppression without a significant DA + Dex-versus-Dex difference. Thus, the clearest long-term combination-associated effect remained *MMP13* suppression. Furthermore, the observed ΔHSA score and Dex-normalized relative-expression analysis further support that this enhancement was not driven solely by variation in baseline expression or unstimulated controls. Together, these data indicate that DA selectively reinforces glucocorticoid regulation of *MMP13* under chronic culture conditions, complementing the acute dose–response interaction observed in the PGE_2_ assays.

Finally, DA interactions at the level of PPARγ activation may underlie our observations. DA, as a selective PPARγ ligand [18], increased nuclear DNA-binding activity, while Dex alone had minimal effect. Dex did not enhance rosiglitazone; however, when DA and Dex were combined, PPARγ activation exceeded the best single-agent response. This ligand-specific interaction is consistent with models in which Dex modulates receptor abundance or chromatin accessibility, and DA stabilizes a transcriptionally favorable conformation. Such cooperative regulation could contribute to gene expression toward pro-resolving and anti-catabolic programs. Importantly, although increased PPARγ DNA-binding activity was directly observed, PPARγ dependency of the transcriptional responses was not established and will require antagonist, knockdown, or equivalent mechanistic studies.

Based on these findings, we hypothesize that DA treatment promotes a “dual intracellular trap” for cAMP and prostaglandins to promote PPARγ activation. It is well established that inhibition of MRP4 can lead to a buildup of intracellular PGE_2_, resulting in conversion to 15-keto-PGE2 by 15-hydroxyprostaglandin dehydrogenase (15-PGDH) [32]. In addition, PGD_2_ may be retained following inhibition of MRP4, which can then be converted to another biologically active metabolite, 15d-PGJ_2_ [33]. Importantly, anti-inflammatory 15-PGDH and 15d-PGJ_2_ molecules can both serve as PPARγ ligands [33,34]. Moreover, a known synergism exists between cAMP and PPARγ, in which cAMP upregulates the expression of transactivation cofactors [35]. Since PPARγ activation has been shown to ameliorate OA in mouse models [36] and downregulate MMP13 in IL-1β-activated human chondrocytes [37], these pathways provide a plausible mechanistic context for the combination responses observed here. Given the central role of MMP13 in structural cartilage loss, this finding suggests that engaging PPARγ-linked pathways may stabilize catabolic control in ways not achievable with corticosteroids alone. Taken together, the observed effects present a model in which DA-mediated inhibition of MRP4 could promote intracellular retention of prostaglandin metabolites and cyclic nucleotides, thereby creating conditions favorable for enhanced PPARγ signaling. However, this framework remains hypothetical and requires direct mechanistic testing.

Viewed together, the PGE_2_ and temporal transcriptional findings support a temporally layered model in which DA complements Dex at different stages of the inflammatory and remodeling response. The combination enhanced acute control of extracellular PGE_2_ and later reinforced selected catabolic and matrix-remodeling responses, most clearly *MMP13* suppression and treatment-associated *COL1A1* suppression. This convergence provides an in vitro rationale for future evaluation of a Dex dose-sparing strategy, but it does not establish dose equivalence, reduced toxicity, or improved matrix preservation because lower-dose Dex + DA was not directly compared with the tested Dex concentration across the long-term endpoints. The dose-sparing interpretation should therefore remain a translational hypothesis for future concentration-response studies that include inflammatory, transcriptional, matrix-production, viability, senescence, and hypertrophic outcomes.

It is important to note that this study has several limitations. For example, the HSC-N and HSC-OA models were each derived from a single human knee donor, with the acute PGE_2_ and prolonged qPCR/PPARγ endpoints evaluated in different donor-specific models. Therefore, the findings do not fully capture donor heterogeneity or establish a single mechanistic sequence in OA chondrocytes. As with other prolonged in vitro chondrocyte models, monolayer expansion and extended culture may promote phenotypic drift, and transcriptional responses do not establish corresponding protein activity, extracellular-matrix preservation, or in vivo efficacy. Accordingly, smaller or isolated transcriptional changes require further functional validation before biological significance can be established. In addition, matrix synthesis, apoptosis, explant validation, and direct mechanistic dependence on MRP4 or PPARγ were also not evaluated. Consequently, these findings should be considered hypothesis-generating. Nonetheless, the convergence of effects across PGE_2_, *MMP13*, and PPARγ supports further investigation of Dex + DA across independent donors, including direct mechanistic testing and evaluation of potential dose-sparing and structural effects.

## 5. Conclusions

In conclusion, these findings identify complementary effects of Dex and DA across distinct inflammatory, transcriptional, and regulatory endpoints in the donor-specific human chondrocyte models studied. The acute PGE_2_ potency findings provide an in vitro rationale for testing whether DA could permit reduced Dex exposure while maintaining inflammatory control, while DA reinforced later Dex-mediated repression of *MMP13*, supporting potential anti-catabolic benefit. Enhanced PPARγ activation further suggests that the combination may influence regulatory pathways in addition to acute inflammatory and catabolic responses. Collectively, these hypothesis-generating observations support further investigation of Dex + DA combinations as a complementary approach with potential to improve inflammatory control, sustain suppression of catabolic gene expression, and enable future evaluation of dose-sparing efficacy.

## Data Availability

The original data presented in this study are openly available in Mendeley Data at https://doi.org/10.17632/x8j4c859k3.1.

## Supplementary Materials

The following supporting information can be downloaded at: https://www.mdpi.com/article/10.3390/biomedicines14092028/s1, Table S1: Experiment-level temporal qRT-PCR treatment contrasts and statistical results for chondrocyte markers; Table S2: Underlying experiment-level ΔCt values for the temporal qRT-PCR analysis; Table S3: Experiment-level assessment of GAPDH Ct across treatment conditions; Table S4: GAPDH-normalized relative expression of chondrocyte markers following treatment with DA, Dex, or DA + Dex. Table S5: Representative OA chondrocyte MTS assay data.
